# Patterns of HIV testing, drug use, and sexual behaviors in people who use drugs: findings from a community-based outreach program in Phnom Penh, Cambodia

**DOI:** 10.1186/s13722-017-0094-9

**Published:** 2017-12-05

**Authors:** Gitau Mburu, Chanrith Ngin, Sovannary Tuot, Pheak Chhoun, Khuondyla Pal, Siyan Yi

**Affiliations:** 1 Division of Health Research, Lancaster University, Lancaster, UK; 2 KHANA Center for Population Health Research, No. 33, Street 71, Phnom Penh, Cambodia; 3 Center for Global Health Research, Touro University California, Vallejo, CA USA

**Keywords:** 90–90–90 targets, HIV testing, HIV risk, People who use drugs, People who inject drugs, Cambodia

## Abstract

**Background:**

People who use drugs are an important priority for HIV programs. However, data related to their utilization of HIV services are limited. This paper reports patterns of HIV testing, drug use, and risk and service perception among people who use drugs. Study participants were receiving HIV and harm reduction services from a community-based program in Phnom Penh, comprised of itinerant peer-led outreach and static drop-in centers.

**Methods:**

This was a mixed-methods study conducted in 2014, comprising of a quantitative survey using a structured questionnaire, followed by two focus group discussions among a sub-sample of survey participants. Participants were recruited from hotspots in five HIV high-burden communes using a two-stage cluster sampling method. Quantitative descriptive analyses and qualitative thematic analyses were performed.

**Results:**

This study included 151 people who use drugs with a mean age of 31.2 (SD = 6.5) years; 77.5% were male and 39.1% were married. The most common drugs used were methamphetamines (72.8%) and heroin (39.7%), and 38.0% injected drugs in the past 3 months. Overall, 83.3% had been tested for HIV in the past 6 months, of whom 62.5% had been tested by peers through community-based outreach. However, there were ongoing HIV risks: 37.3% were engaging in sex on drugs, only 35.6% used a condom at last sexual intercourse, and 10.8% had had a sexually transmitted infection in the last 6 months. Among people who reported injecting drugs in the past 3 months, 27.5% reported re-using needles/syringes. Almost half (46.5%) perceived themselves as being at lower risk of HIV compared to the general population. Qualitative results contextualized the findings of low perception of HIV risks and suggested that although services were often unavailable on weekends, at night, or during national holidays, peer-led community-based outreach was highly accepted.

**Conclusions:**

A peer-led community-based approach was effective in reaching people who use drugs with HIV and harm reduction interventions. To mitigate ongoing HIV risks, expanding access to combination prevention interventions and implementing strategies to enable people who use drugs to objectively assess their HIV risks are required. Additionally, community-based programs should collect data along the care continuum, to enable decentralized tracking of progress towards 90–90–90 goals at local levels.

## Background

Prompt HIV diagnosis, timely linkage to care, early treatment, retention in care, and sustained viral suppression are all essential for optimal impact of antiretroviral therapy (ART) in reducing morbidity and mortality from human immunodeficiency virus (HIV) [1–4]. The time-sensitive sequencing of clinical and operational events that commence with an HIV diagnosis and end with long term viral suppression epitomize the continuum of HIV care [5].

However, significant loss to follow-up of people living with HIV occurs in the various steps between HIV testing and viral suppression [6, 7], resulting in an undesirable cascade [5]. Hence, a paradigm shift focusing on proportions of people living with HIV that are tested, linked to care, retained in care, adherent to ART, and achieving sustained viral suppression is increasingly framing outcomes of HIV care. The UNAIDS 90–90–90 targets, which aim to ensure that 90% of all people living with HIV know their status, 90% of all diagnosed people living with HIV receive ART, and 90% of all persons receiving ART achieve viral suppression [8], capture this paradigm shift.

Achievement of these global goals will depend on their attainment among all people living with HIV and key populations including people who use drugs [9]. People who use drugs constitute one of the most vulnerable populations to HIV. Unsafe drug use accounts for a third of newly diagnosed HIV infections outside sub-Saharan Africa [10], and 10% of all HIV infections globally [11]. Drug use is particularly widespread in Asia [12], and its impact on HIV is increasing [13]. In Cambodia, where the national HIV prevalence is 0.3% among the general population, people who use drugs are disproportionately affected, with a prevalence of 4.0% among people who use non-injecting drugs and 24.8% among individuals who inject them [14].

Given the importance of people who use drugs in the global HIV epidemic, it is essential that early testing, linkage, retention, and viral suppression are achieved among these groups. Currently, however, very few countries are well poised to attain the 90–90–90 targets in people who use drugs by 2020. A recent review revealed a significant lack of data related to HIV care continuum among people who use drugs. In the few countries where data are available, care outcomes among people who use drugs are significantly below 90–90–90 targets [9]. Individual studies have reported that people who use drugs have low rates of ART coverage [15], HIV testing [9], adherence [16], retention [17], and viral suppression [9, 17]. Although contextual differences exist, these poor outcomes are generally caused by a range of individual, social, legal, health system, and other structural barriers [13, 18, 19]. In addition, effective models for promoting the widespread reach and entry of people who use drugs into HIV care are generally limited globally [20].

Cambodian national response has placed an emphasis on key populations, including people who use drugs, in order to achieve the 90–90–90 target and eliminate new HIV infections under Cambodia 3.0 [21]. A recent review reported that, of the estimated 72,607 people living with HIV, 60,336 (83%) are diagnosed, 54,769 (75%) are receiving ART, and 50,935 (70%) are virally suppressed. As such, Cambodia is one of the few countries on track to achieve 90–90–90 targets [22]. However, this review did not evaluate care continuums for specific populations (e.g., key populations), which—authors assert—would more directly inform programming strategies for these populations [22].

In Cambodia, there are still gaps in the data and strategic information on the HIV situation and response, in particular among key affected populations [23]. The national estimated number of people who use non-injecting drugs and people who inject drugs was 13,000 and 1300, respectively [14]. However, precise 90–90–90 estimates among these populations are unavailable. Like many other countries, strategic information in each step along the continuum for people who use drugs is limited [14]. One of the five key objectives of the National Strategic Plan on Harm Reduction is strengthening the strategic information base [23].

In this paper, we report patterns of HIV testing, drug use, and sexual behaviors among people who use drugs who were receiving HIV and harm reduction services from a community-based HIV program in Phnom Penh. We also report their self-perception of HIV risk and views regarding community-based services they were receiving, in order to provide information that could improve services for this population. We conclude this paper with a brief discussion of the potential implications of our findings in relation to 90–90–90 targets in Cambodia.

## Methods

### Study design

This was a mixed-methods study that was composed of a large quantitative cross sectional survey, with a smaller qualitative component comprising focus group discussions (FGDs).

### Study settings

The research was conducted among people who use drugs in Phnom Penh, who were in contact with a community-based program known as Sustainable Action against HIV and AIDS in Communities (SAHACOM). SAHACOM was implemented over a 5-year period ending in December 2014, focusing on key populations [24, 25]. The project was implemented by KHANA, a leading HIV non-governmental organization working through numerous community-based organizations (CBOs).

The project utilized a community-based approach to empower and create ownership of the project among key populations, including people who use drugs. The model was composed of itinerant peer-based outreach, and static drop-in centers. Through this model, key population peers were hired by CBOs to provide HIV and harm reduction services to people who use drugs, including HIV education, condoms, clean needles, and syringes. Through peer involvement, the model transformed people who currently or formerly used drugs from passive service recipients to active providers of services [26].

Besides providing the above services in their own localities, peers referred people who use drugs to the static drop-in centers, which were part of community-level infrastructure, where methadone, harm reduction counselling, peer discussions, personal care, needles, syringes, and condoms, among other commodities, were provided. In addition, people who use drugs in contact with the outreach teams were encouraged to form and attend peer discussions at drop-in centers for education purposes.

At the end of the implementation period, data were collected to assist in evaluation of the project. The purpose of the evaluation was to understand the HIV testing and prevention needs of the participants, and their perception of the SAHACOM services. Mixed methods were used to allow contextualization of quantitative survey data. This is a widely used approach of linking quantitative and qualitative data within implementation research [27, 28].

### Participant selection

By the time the SAHACOM was implemented, the estimated total number of people who use drugs in Phnom Penh was 4188; of these 1068 injected drugs. In Phnom Penh, SAHACOM was implemented in 33 communes with high concentration of people who use drugs out of 96 communes in the city. Although accurate general population size for these communes was not available, strategic information related to HIV suggested that these 33 communes covered approximately 1969 people who use drugs, of whom 353 injected drugs. A two-stage cluster sampling method was used to select participants for the quantitative component. At the first stage, the research team identified five communes with at least 20 people who use drugs to be included in the study. A list of ‘hotspots’ where people who use drugs and congregated was made based on geographical clustering of high risk behaviors as is practiced elsewhere [29]. At the second stage, all people who use drugs in the selected hotspots were approached and invited to participate in the study, and were screened for eligibility.

**Fig. 1:**
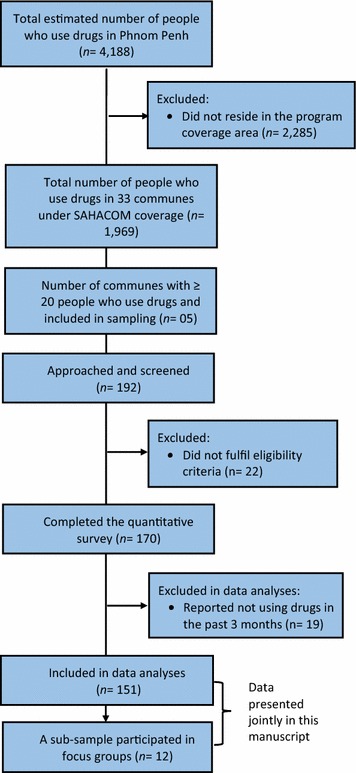
Participant selection process

Potential participants were included in the study if they: (1) were at least 18 years of age, (2) had used any form of illicit drugs in the past 3 months, (3) were able to present themselves on the day of the interview, and (4) were able to provide informed consent to participate in the study. Of the 192 people screened, 22 were excluded, leaving 170 eligible participants. For the qualitative component, a convenience-based subsample was selected from the primary participants in the quantitative phase. At the end of quantitative data collection, participants were invited to take part in focus group discussions based on interest, convenience, availability, and willingness to consent. The participant selection process is illustrated in Fig. 1.

### Study tools development

The quantitative questionnaire was developed in English, translated into Khmer, back-translated to English, and pretested to ensure that it was culturally suitable and clearly understandable. Standardized questions were adapted from previous studies and Demographic and Health Surveys [14, 30, 31] to document participants’ socio-demographic characteristics (e.g. age, sex, marital status, education, occupation, income, residence, and mobility), drug use characteristics (e.g. age of initiation, frequency, method and types and circumstances of drug use, sharing of needles or syringes, as well as history of drug-related arrests and voluntary rehabilitation or incarceration/imprisonment), HIV-related characteristics (e.g. prior HIV testing, venue of HIV testing and counselling, and sources of HIV education), sexual behaviors (e.g. sexual activity, number of sex partners, consistency of condom use, history of STI symptoms, whether STI treatment was sought, and nature of facility from which treatment for STI was sought), and self-perceived risk of HIV. To get a sense of participants’ self-perceived risk of HIV, participants were asked to indicate whether they thought they were at lower, same, or higher risk, relative the general population.

Similarly, a topic guide for qualitative focus groups was developed in English, translated into Khmer, back-translated to English, pretested, and adjusted accordingly following piloting. The objective of the qualitative component was to contextualize and provide insights to the quantitative findings. The focus group topic guide questions were open ended and explored participants’ drug use behaviors, HIV testing experiences, sexual behaviors, perceived risk of HIV, and perception of services they were receiving through the SAHACOM.

### Data collection

Data were collected by researchers in private rooms in drop-in centers. Before data collection, researchers were provided with an overview of the study objectives and trained on the questionnaire filling techniques including error checking, focus group facilitation, privacy assurance, confidentiality, and data protection. Questionnaires were administered face-to-face to 170 participants, each lasting 30–45 min. At the end of survey questionnaires, participants were invited to participate in the focus groups.

Interested and consenting participants were provided with appointment schedules to attend the discussions. Invitation to take part in the focus groups was not based on types of drugs used, as long as participants had participated in the quantitative survey. In total, two focus group sessions, each with six participants, were conducted on the premises of CBOs. Recruitment for the focus groups was guided by saturation of themes, rather than by pre-specified sample size as recommended in qualitative studies [32]. At the end of the second focus group session, it was clear that additional discussions would not yield further insights and recruitment was halted. The focus groups lasted 45–60 min, were tape-recorded, and were conducted in the local Khmer language.

### Data analyses

Quantitative data were coded and entered into a computer using Epi Data version 3 (Odense, Denmark). Double data entry was performed to limit errors. Logic checks were performed, and 19 participants who reported that they had not used any illicit drugs in the past 3 months were excluded from the analyses. Summary of frequencies for nominal and ordinal variables was documented. Descriptive analyses were used to compute means for continuous variables. SPSS version 20.0 (IBM Corporation, New York, USA) was used for all data analyses.

Qualitative audio data were transcribed and transcripts translated to English from Khmer and imported into NVivo (QSR International). Thematic data analysis [33] was then conducted inductively within the original aims of the study, in order to provide context for the quantitative findings regarding sexual and injecting behaviors, nature of services received by participants, and participants’ perception of those services. Nodes were created, and populated with appropriately labelled codes. Codes were populated with textual segments refined using constant comparison approach [34], and then categorized based on similarities to generate overarching themes [32, 33]. Verbatim quotes were extracted from participants’ transcripts and displayed in the text to aid transparency of interpretation as recommended [35]. Qualitative text and quotes were presented alongside quantitative data in order to contextualize or explain survey findings in an integrated fashion [36].

### Ethical considerations

This study was approved by the National Ethics Committee for Health Research (No. 082NECHR). Verbal consent was obtained from each participant. Before consenting, participants were informed of their right to discontinue their participation at any time, without any consequences. Names and contact information of the researchers were provided to participants for use in case they had any questions. Identifiable data were not collected on questionnaires; instead, unique codes were utilized in order to protect confidentiality of participants. Participant were compensated approximately US$5 for their time and transport to participate in the study.

## Results

### Socio-demographic characteristics

In total, 151 people who use drugs participated in the survey, whose mean age was 31.2 (SD = 6.5) years; a subsample of whom (*n* = 12) participated in the focus groups. Of the total participants, 77.5% were male; 39.1% were married, 40.4% were single, and 20.5% were either divorced or widowed. In addition, 14.6% were unemployed, and the sample was generally non-migratory, having lived in their current locations for a mean of 19 years (Table 1).

**Table 1:** Socio-demographic characteristics of participants

Variables	Number (%)
Mean age (years, ±SD)	31.2 ± 6.5
Gender	
Male	117 (77.5)
Female	34 (22.5)
Marital status	
Married	59 (39.1)
Divorced, widow	31 (20.5)
Single	61 (40.4)
Mean years of schooling completed (±SD)	5.2 ± 4.1
Main occupation	
Unemployed	22 (14.6)
Laborer	33 (21.9)
Self-employed business	45 (29.8)
Office worker	10 (6.6)
Other	41 (27.2)
Monthly income (US$, ±SD)	313.7 ± 601.0
Currently living with:	
Parents	41 (27.2)
Spouse/partner	60 (37.7)
Friend/colleagues	19 (12.6)
Siblings/relatives	14 (9.3)
Alone	17 (11.3)
Mean length of residency in Phnom Penh (in months, ±SD)	233.6 ± 149.3

*SD* standard deviation

### Drug use practices

As shown in Table 2, 38.0% of the total participants had injected drugs in the past 3 months. On average, participants started using drugs at the age of 21.3 (SD = 6.5) years, and had used drugs for the past 8.4 years. The most common drugs used were amphetamines (72.8%) and heroin (39.7%). Although most participants used drugs either with friends (54.4%) or alone (31.1%), a notable proportion (14.5%) used drugs with their sexual partners. More than half (55.3%) of the overall sample had been referred to a rehabilitation center in their lifetime, and 21.9% had been sent to the center in the past 12 months. A third (31.3%) had ever been incarcerated/imprisoned due to drug possession and other offences.

**Table 2:** Drug use practices among the study participants

Variables	Number (%)
Type of drugs used in the past 3 months	
Methamphetamines	110 (72.8)
Heroin	60 (39.7)
Other (ecstasy, sniffed glue, marijuana, etc.)	5 (3.3)
Mean duration of drug use (months, ±SD)	103.8 ± 68.7
Mean age at first time of illicit drug use (years, ±SD)	21.3 ± 6.5
Frequency of illicit drug in the past 3 months	
Everyday	77 (51.3)
Almost everyday	21 (14.0)
A few times a month	24 (16.0)
A few times a week	27 (18.0)
People with whom you used drugs the last time	
Alone	47 (31.1)
Friend	82 (54.4)
Sexual partner/sweetheart	22 (14.5)
Reason to led you try illicit drugs for the first time	
I tried it by myself	45 (29.8)
Someone gave it to me or forced me to use it	6 (4.0)
Tried it with friends	81 (53.6)
Other	19 (12.6)
Injected drugs in the past 3 months	57 (38.0)
Used a used needle when injected drugs the last time (*n* = 57)	14 (27.5)
Shared needles at last drug injection (*n* = 57)	8 (15.7)
Perceived that needle/syringes are easy to find (*n* = 57)	50 (87.7)
Have been arrested for drug use or trafficking	70 (46.4)
Have ever been sent to a drug rehabilitation center	83 (55.3)
Have been sent to a rehabilitation center in the past 1 year	33 (21.9)
Had ever been incarcerated/imprisoned	50 (33.1)
Main cause of most recent incarceration/imprisonment	
Drug use or possession	11 (22.4)
Drug-related crimes (e.g. theft)	31 (63.3)
Other crimes (non-drug related)	7 (14.3)

Less than a third (27.5%) of participants who injected drugs in the past 3 months reported that they had used a needle that had been used by someone else the last time they injected. Contextualizing this finding a participant explained that, “we *stopped sharing syringe…we try to prevent it*” (respondent # 1, focus group 1), while another asserted that, “*we take a methadone regularly, stopped injecting and stopped sharing syringes*” (respondent # 4, focus group 1). Participants mentioned that peer educators were an important source of syringes for the participants. Besides commodity supplies, participants relied on peer educators for “*transportation service to get methadone*” (respondent # 4, focus group 1).

However, participants raised concerns regarding a lack of supplies on weekends or other days when national holidays and ceremonies were being held. One participant asserted that, “*on Saturday and Sunday, if we don’t have, we need to buy*” (respondent # 6, focus group 1), because as explained by another participant, “*they just don’t work at weekends but Monday to Friday they come to meet people at local*” (respondent # 5, focus group 1). Participants also explained that re-using or sharing needles was common at night when getting syringes or needles was limited:

- “*Sometimes, people inject around 12 or 1 at night, so during that time no one comes to provide you syringes or needles*” (respondent # 2, focus group 2).

Despite this gap in service provision, participants asserted the value of outreach suggesting that, “*they do not come and provide services all the time; however, their services reduce the cost of our expenses*” (respondent # 2, focus group 1).

### HIV counselling and testing

As shown in Table 3, 96.0% had ever been tested for HIV in lifetime, and 83.3% had received an HIV test in the past 6 months. In addition, 97.2% had received counselling during their most recent HIV test. A majority (62.5%) of the HIV testing was delivered through community- or peer-initiated counseling and testing, with a minority (9.0%) having been provided through voluntary counseling and testing centers. In addition, 86.6% had received HIV education in the past 12 months, and although most participants received this information from several different sources, the most common source of HIV education was peer educators or outreach workers (89.2%). These qualitative data suggested that community-based outreach program was an important source of HIV education, counselling and testing which was relatively acceptable to participants.

**Table 3:** Access to HIV counselling, testing, and education

Variables	Number (%)
Ever tested for HIV	144 (96.0)
Tested for HIV in the past 6 months (*n* = 144)	120 (83.3)
Place for last HIV testing (*n* = 144)	
Voluntary counseling and testing center	13 (9.0)
Community/peer-initiated testing	90 (62.5)
Public health center/clinic/hospital	31 (21.5)
Private clinic/hospital	10 (6.9)
Received result of the most recent HIV test (*n* = 144)	143 (99.3)
Received counseling for the last HIV test (*n* = 144)	139 (97.2)
Received HIV education in the past 12 months	129 (86.6)
Source of HIV education in the past 12 months (*n* = 129)	
Mass media (television/radio/newspaper)	60 (46.2)
Poster/billboard	34 (26.2)
Peer educator or outreach worker	116 (89.2)
Voluntary counseling and testing session	28 (21.5)
Health staff	43 (33.1)
Self-perceived level HIV risk compared to the general population	
Higher	52 (36.1)
Same	19 (13.2)
Lower	67 (46.5)
Don’t know	6 (4.2)

*HIV* human immunodeficiency virus

Contextualizing these findings, qualitative focus groups supported the view that HIV testing and education provided by peers and outreach workers were acceptable to participants. Referring to these peers and outreach workers, a focus group participant affirmed that, “*they help us to know that we are reactive HIV…. if we are HIV positive we go to get ART*” (respondent # 1, focus group 2). This participant further commented that, “*the organization always comes to educate us.*” (respondent # 1 focus group 2), while others contrasted access to testing in health facilities by asserting that they found outreach services easily accessible “*because they come two times per day*” (respondent # 6, focus group 2).

### Sexual behaviors

As shown in Table 4, 37.3% of the participants reported having sex while intoxicated with drugs. In addition, 10.8% had been diagnosed with an STI in the past 6 months. At the same time, 41.1% reported having been involved in sexual intercourse in the past 3 months, and only 35.6% reported using a condom in their last sexual intercourse act.

**Table 4:** Sexual behavior among the study participants

Sexual behaviors	Number (%)
Diagnosed with an STI in the past 6 months	16 (10.8)
Sought treatment for most recent STI symptom (*n* = 16)	2 (11.1)
Facility where the most recent STI was treated	
Public health center/hospital (*n* = 2)	1 (50.0)
Non-governmental clinic/hospital (*n* = 2)	1 (50.0)
Had sexual intercourse in the past 3 months	59 (41.3)
Used condom in last sexual intercourse (*n* = 59)	21 (35.6)
Had sexual intercourse when intoxicated (*n* = 59)	22 (37.3)

*STI* sexually transmitted infections

Qualitative data provided additional insights regarding the inconsistent condom use. In particular, narratives from the focus groups suggested that participants did not believe that they needed to use condoms with their spouses because they trusted them. Asked why they were not using condoms, participants responded by saying that, “*I trust my wife*” (respondent *#* 6, focus group 1), “*Yes, I believe my wife*” (respondent *#* 5, focus group 1), among other similar responses.

### HIV risk perception

As shown in Table 3, a majority of participants (56.5%) viewed themselves to be at the same or lower risk of HIV infection compared to the general population. Although there were other reasons for not using condoms, such as a “*desire to have a child*” (respondent *#* 3, focus group 1), the finding of low condom use among the sample is relevant as it corroborates the low self-perception of HIV risk among the participants. While responding to a question regarding whether he thought he was at risk of HIV, a participant stated that, “*I have only my wife, I don’t have any other partners*” (respondent *#* 4, focus group 1), showing that participants viewed HIV risk as external to themselves and their drug-using behaviors.

Further exploration of this issue in the focus groups suggested that participants believed that their increased knowledge was contributing to their protection from HIV, and they were no longer at high risk of HIV. When asked whether they thought their group was a high-risk group, participants contrasted the previous lack of knowledge with their current situation, stating that:

- “*Firstly, we didn’t have a support from NGOs, secondly we don’t have a counseling from NGOs and that made us lack knowledge about health care or prevention, so it made us a high risk group*” (respondent # 5, focus group 2).

This participant, who belonged to a specific peer discussion group of drug users in one of the drop-in centers went on to state that:

- “*Now, I think my group is a low-risk group, because we have a lot of knowledge from NGOs that come to educate us*” (respondent # 5, focus group 2).

These sentiments were supported by others who emphasized the changes in risks occurred due to the changes in sexual and injecting behaviors, primarily due to increased supply of harm reduction commodities. In a typical response regarding why they thought they were at low risk of HIV, a participant stated that, “previously, we didn’t have condoms to use and didn’t have any NGOs coming to provide them” (respondent *#* 2, focus group 2). Others also referred to condoms, stating that their HIV risk was reduced “because we have NGOs who taught about using condoms; now we know how to use it, especially to prevent STIs” (Respondent *#* 6, FGD 1).

Apart from availability of condoms, few participants also referred to availability of clean syringes as giving them a sense that they were at low risk. For instance, one participant mentioned that, “as for now, we do not have any disease because we use new syringes” (respondent *#* 1, focus group 1). As can be noted from the above excerpts, participants felt that, apart from knowledge per se, their use of preventive interventions was protecting them from HIV. Within these discussions, it emerged that the use of these comodities was enhanced by their free provision:

- “*Now we have NGOs to provide them, it isn’t like before when we didn’t have and we needed to buy*” (respondent # 4, focus group 1).

## Discussion

This paper describes important information regarding access to HIV testing among people who use drugs. The study reports a high level of access to HIV testing in the context of ongoing injecting and sexual risks, and in a country where a quarter of people who inject drugs (24.8%) are HIV infected [14]. The high proportion of participants who had ever been tested (96.0%), or were tested in the last 6 months (83.3%) is notable given the generally low rates of testing among people who use drugs reported from other settings [9, 37, 38].

A number of factors may have contributed to this observation, the most likely being the model of community-based outreach, which embraced the deployment of community support volunteers, peer facilitators, and peer educators to provide services. The majority of those who were tested accessed testing from these peer providers rather than voluntary counseling and testing centers. The proportion of participants tested for HIV was high despite the perceived low risk of HIV. This approach may also have mitigated transport, administrative, attitudinal, and trust-related barriers which deter testing of people who use drugs in conventional health facilities [39, 40].

The utilization of drop-in centers and community-level infrastructure to provide methadone, personal care, counselling and education, needles, syringes, condoms, and other commodities may have synergistic effects on the uptake of these HIV and harm reduction interventions. Qualitative findings suggested that methadone was perceived as providing opportunities to reduce drug injecting, while increasing uptake of clean needles and syringes. These findings endorse the use of methadone to mitigate risks [41, 42], and the use of community-based outreach model for providing integrated HIV and harm-reduction services [43]. Other studies have shown that the use of socially familiar infrastructure, such as community-based drop-in centers can act in synergy with the use of peer counsellors or educators or outreach workers to enhance HIV testing, entry into HIV care, and uptake of harm-reduction interventions [20, 39, 44, 45].

In spite of the close contact with peer-based outreach services, our findings highlighted ongoing risks of HIV infection among the study sample. Study participants believed that their knowledge of HIV and adoption of safer injecting practices reduced their risk of HIV. While this might be the case, participants were still exposed to significant risks. The low perceived level of risk, combined with a low rate of condom use among those who are sexually active, engagement in sex while intoxicated, and the occasional sharing of needles, operate together to escalate potential for HIV infection.

These enduring risks suggest a need for additional prevention interventions and delivery strategies tailored to participants’ risk profile. For instance, despite the advantages of peer outreach model, a lack of outreach services over weekends, nights, and bank holidays increased risky injecting behaviors. While firm conclusions may not be made about the impact of these gaps, these findings suggest a need for innovative methods such as self-dispensing or vending machines for needles and syringes for participants who encounter these temporal shortages. On the other hand, PrEP might be useful in situations where condom use is intended but does not occur, which is relevant for couples, given that almost half of the participants were in stable relationships yet condom use with spouses was sub-optimal.

In addition, a sixth of participants used drugs with their sexual partners, further lending weight to other calls that testing and prevention services should be extended to sexual partners of people who use drugs [46]. Use of methamphetamine and other drugs during sex, colloquially termed ‘chemsex,’ is associated with higher sexual HIV transmission risks [47, 48], and requires to be addressed through specifically tailored information. Given the noted disconnect between perceived risks and actual HIV risk behaviors, further expansion of the scope of HIV education is required to enable participants objectively assess and understand their HIV risks. For instance, models of estimating actual risks [49] could be incorporated into educational efforts.

### Limitations of the study

The study involved participants who were already in contact with peer outreach. It is therefore possible that this recruitment bias may have affected our results related to participants’ uptake of HIV testing and perception towards peer-led community-based outreach. A reason for the finding that participants held the perception that they had lower HIV risk compared to the general population may have related to their recent HIV testing. Moreover, the proportion of participants who were already HIV positive was not known as participants were not required to divulge their HIV status. Consequently, the relationship between recent test results and risk perception could not be explored in this study. All participants were recruited in Phnom Penh, limiting generalizability to other people who use drugs nationally. Participants in the focus groups were selected based on convenience, which may exacerbate this selection bias. Despite application of eligibility criteria designed to retain participants that were currently using drugs, a small minority reported they had not used any form of illicit drugs in the past 3 months. More broadly, the methodology used in this study largely depended on participants self-reporting, and some participants skipped some questions, which may have introduced further limitations.

### Implications for future research

Despite these limitations, our findings are consistent with research in Cambodia and elsewhere showing the effectiveness of community-based outreach in reaching people who use drugs [20, 50]. While this study reports on uptake of HIV testing, downstream data on linkage and retention on ART was unavailable, yet are central in the care continuum. Besides, the extent to which the program contributed to achievement of the first 90 in the 90–90–90 targets cannot be deduced because of lack of denominators and prior rates of testing. This is due to lack of local level strategic information regarding numbers of people at risk. Nationally, past efforts predominantly focused on increasing HIV testing, with significant limited focus on the continuum on local levels [23]. This was certainly the case in 2014 when this study was conducted [23].

However, the Health Sector Strategic Plan IV (2015–2020) includes strategies for improving the strategic information base as one of its cross-cutting strategy. Generation of localized data will enable programs monitor their contributions to 90–90–90 targets better, within their catchment areas. In addition, focus on the care continuum is central to the relatively recent approach—known as Boosted Continuum of Prevention to Care and Treatment—which aims to identify and reach new infections, ensure they are brought into care, and are retained in ART. This approach is targeted specifically to areas where new infections are occurring, among key populations, and should therefore be central to future community-based outreach programming and research.

## Conclusions

The findings of this study demonstrate that utilization of peer outreach and community infrastructure familiar and close to people who use drugs such as drop-in centers could contribute to increasing uptake of HIV testing and other harm reduction interventions among these populations. Although the uptake of testing, needles, and syringes was high in this study, outstanding HIV risks were identified. Additional strategies are required to ensure that people at risk are enabled to have an objective understanding of their HIV risks and vulnerabilities of people who use drugs are mitigated in the study context.
